# The inside of me: interoceptive constraints on the concept of self in neuroscience and clinical psychology

**DOI:** 10.1007/s00426-021-01477-7

**Published:** 2021-05-28

**Authors:** Alessandro Monti, Giuseppina Porciello, Maria Serena Panasiti, Salvatore Maria Aglioti

**Affiliations:** 1 Sapienza, Università di Roma and CLNS@Sapienza, Istituto Italiano di Tecnologia, Rome, Italy; 2 IRCCS Fondazione Santa Lucia, Rome, Italy; 3 Dipartimento di Psicologia, Sapienza, Università di Roma, Rome, Italy

## Abstract

Humans are unique in their ability to think about themselves and carry a more or less clear notion of who they are in their mind. Here we review recent evidence suggesting that the birth, maintenance, and loss of the abstract concept of ‘self’ is deeply tied to interoception, the sense of internal physiological signals. Interoception influences multiple facets of the self-concept, cutting across its material, social, moral, and agentive components. Overall, we argue that interoception contributes to the stability of the self-concept over time, unifying its layers and constraining the degree to which it is susceptible to external influences. Hence, the core features of the self-concept are those that correlate more with inner bodily states. We discuss the implications that this may have for theories of embodied cognition as well as for the understanding of psychiatric disorders in which the concept of self appears fragmented or loose. Finally, we formulate some empirical predictions that could be tested in future studies to shed further light on this emerging field.

## Introduction

The concept of self, i.e. who you think you are, encompasses the sum total of the features that you affirm of your own being and deny of all other beings. However, the number and type of these distinctive features can vary widely. Evidence from classical psychometric tests, such as the Twenty Statements Test (Kuhn & McPartland, 1954), suggests that people define themselves by describing their physical appearance (*I am six feet tall; I am dark-haired*), telling their biographical details (*I am the son of a surgeon*), stating their social roles (*I am married; I am a pianist*), specifying their political, ethnic, or religious group (*I am a conservative; I am Jewish*), listing their virtues and vices (*I am honest; I am lazy*), or locating their place in the grand scheme of things (*I am a human; I am a rational animal*). Thus, the self is a multifaceted construct, encompassing a broad set of information that ranges from the mundane and material to the social and spiritual. At the same time, the self is indeed a concept and not a motley array of disconnected details, since that set of information consistently points to a unique individual across a diverse range of circumstances. More specifically, the self-concept is an *abstract* concept, as it exhibits the two peculiar marks of abstractness: it does not have a single and perceptually bounded kind of objects as referent, and it can be applied to a variety of complex situations (Borghi et al., 2018).

Abstract concepts have long been considered a challenge for theories of embodied cognition, as they seem to lack a clear, unambiguous bodily counterpart. However, recent studies on abstract concepts suggest that the birth, maintenance, and loss of these concepts in the human mind can be in fact correlated with bodily states (Borghi et al., 2018; Connell et al., 2018; Villani et al., 2021). As a case in point, in this review, we aim to show how a highly symbolic notion like the sense of self is shaped by radically corporeal dimensions like those carried out by interoception, i.e. the perception of physiological signals coming from the inner parts of the body, such as heartbeats, breaths, gastric contractions, and other cues that inform us about our homeostatic condition (Craig, 2002). The upshot is that internal bodily signals exert a pervasive, stabilising influence on three of the four layers of James’s (1890) classical partition of the self: not just the *material self* (the concept of myself as a material being), but also the *social self* (the concept of myself as a member of society) and even the *spiritual self* (the concept of myself as a moral person). Furthermore, we will review the preliminary evidence suggesting that internal bodily states are also linked to the fourth Jamesian layer, namely, the *pure Ego* (the concept of myself as a thinking and acting subject). Across all levels, we will also assess what happens to the concept of self when interoceptive signals are not processed in a proper way. Finally, we will discuss the implications of these interoceptive constraints for theoretical accounts of the self-concept and formulate some empirical predictions that could be tested by future studies in the field.

## Interoceptive constraints on the material self

If one had to make an inventory of the features that uniquely define one’s own being, in all likelihood one’s own body would feature prominently on the list. We spontaneously include our body in our concept of self because we feel that the body is always with us, at least when we are sane and awake. Thus, the body is not just an object among many others; rather, it is the key component of our *material self* (James, 1890). This feeling of intimacy with one’s own body is variously termed as corporeal awareness (Berlucchi & Aglioti, 1997, 2010; Critchley, 1979), embodiment (Longo et al., 2008) or bodily self-consciousness (Blanke et al., 2015). It can be thought of as a three-pronged construct, as someone who is aware of their body is aware of *having* a body (sense of body ownership), of *controlling* its movements (sense of body agency) and of *dwelling* in it (sense of body location).

Research on neurological disorders (Blanke & Arzy, 2005; Heydrich & Blanke, 2013; Ronchi et al., 2018) and on bodily illusions like the rubber hand (Botvinick & Cohen, 1998), the full-body illusion (Lenggenhager et al., 2007), the body swap illusion (Petkova & Ehrsson, 2008) and the enfacement illusion (Paladino et al., 2010; Sforza et al., 2010; Tsakiris, 2008) implies that the body becomes part and parcel of one’s material self when the appearance of the body, the position of its parts, and other cues occur at the same time and in the same place so that the brain can integrate them in an efficient manner (Blanke et al., 2015). In principle, these cues can come both from outside and from inside the body. Classical studies on corporeal awareness chiefly focused on exteroceptive signals, from touch to vision: that is, researchers investigated how various features of exteroception determined specific changes in the awareness of one’s own body, both in its constituent parts and as a whole. However, interoception, i.e. the sense of the physiological condition of the body (Craig, 2002), is increasingly thought to have an important role in shaping corporeal awareness, too (Craig, 2009; Critchley & Harrison, 2013; Herbert & Pollatos, 2012; Park & Tallon-Baudry, 2014).

To date, research trying to ascertain the impact of interoceptive signals on the material self has been hampered by the fact that these signals are extremely diverse and difficult to record, but some significant progress has been made. For example, a series of experiments applied an interoceptive twist to the standard bodily illusions. Tsakiris et al. (2011) found that the degree of susceptibility to the illusion correlates with individual levels of interoceptive accuracy, i.e. how well participants objectively detect inner bodily signals at the conscious level, as in the heartbeat counting task (Schandry, 1981): the worse their performance, the more likely they were to self-report that the rubber hand was theirs. In a similar vein, people with low interoceptive accuracy are more prone to include the face of another person in their self-face representation (Tajadura-Jiménez & Tsakiris, 2014). Although these studies tantalisingly suggest the existence of a closed loop between interoception and corporeal awareness, they did not manipulate either the physiological signals giving rise to interoception or the perceptual or symbolic representation of these signals in the minds of the participants.

Other research groups made a step further by making a virtual rubber hand (Suzuki et al., 2013) or a full virtual body (Aspell et al., 2013) or a face (Porciello et al., 2016; Sel et al., 2017; but see Porciello, Bufalari, et al., 2018) flash either in sync or out of sync with the participant’s heartbeat. Their results suggested that visuo-cardiac synchrony generally boosts embodiment. However, one may wonder whether cardiac signals can be considered an embodiment factor also in ordinary ecological circumstances, in which heartbeats are perceived only faintly and transiently. An elegant study by Park et al. (2016) answered the question in the affirmative. Combining electrocardiography, electroencephalography, and virtual reality, they discovered that heartbeat-evoked potentials (HEPs) originating from the posterior cingulate cortex are linked to changes in corporeal awareness induced by the full-body illusion.

This landmark result spurred the quest for other forms of coupling between visceral signals and bodily self-consciousness beyond the cardiac domain. Rebollo et al. (2018) did find that also the electrical oscillations coming from the interstitial cells of Cajal in the stomach can explain a sizeable degree of fluctuation in the resting BOLD signal of a cluster of brain areas they termed the ‘gastric network’. Although the authors propose that this gastro-cerebral coupling contributes to the representation of the bodily self, further research is needed, since this study relied on a no-task, resting state paradigm and the gastric network may simply act as a homeostatic circuit sensitive to hunger cues (Porciello, Monti, et al., 2018).

Other lines of research on corporeal awareness targeted physiological signals straddling the boundary between interoception, proprioception and exteroception. For example, pleasant touch, which is mediated by ‘interoceptive’ C tactile afferents (Björnsdotter et al., 2010), is more effective than neutral touch, which is coded by ‘exteroceptive’ Aβ afferents, in giving rise to the rubber hand illusion (Crucianelli et al., 2013; Lloyd et al., 2013; van Stralen et al., 2014). However, both kinds of touch are equally effective drivers of the full-body illusion (Carey et al., 2021). Recent studies have also elucidated the embodying power of another multisensory cue, respiration. In a variation of the cardiac full-body illusion (Aspell et al., 2013), participants report a higher degree of self-identification with virtual silhouettes flashing in sync with their breathing (Adler et al., 2014; Allard et al., 2017). In the new ‘embreathment’ illusion, experimental subjects show higher ratings of body ownership and agency towards a full-fledged virtual body that inspires and expires as the real body of each participant, compared to an avatar that displays an inverted, anti-phase breathing pattern (Monti et al., 2020).

Furthermore, the strength of the embreathment illusion is moderated by individual levels of interoceptive accuracy and sensibility: those who objectively detect bodily signals with more accuracy or subjectively report to pay more attention to them are also those who are less swayed by the illusion itself (Monti et al., 2020). Symmetrically, patients with eating disorders are both worse than healthy controls at feeling their cardiac signals (Jenkinson et al., 2018; Pollatos et al., 2008) and more likely to embody a rubber hand (Eshkevari et al., 2012; Keizer et al., 2014). In a similar vein, schizophrenia patients are less interoceptively accurate (Ardizzi et al., 2016), are more prone to incorporate a rubber hand than controls (Peled et al., 2000; Thakkar et al., 2011), and display an altered sense of body agency (for an excellent review, see Hur et al., 2014). These convergent findings suggest that inner bodily signals, if properly felt, buttress the material self so that it becomes more stable. Later on, we will see how this could fit in a bigger picture of the relationship between interoception and the whole concept of self.

## Interoceptive constraints on the social self

James (1890) perceptively noted that “if every person we met 'cut us dead,' and acted as if we were non-existing things” they would wound us more than if they physically tortured us. This form of social pain implies that we would deem ourselves incomplete if we did not get any recognition from our mates. Thus, in addition to the material self, we also have a social self: our concept of ourselves includes not just our body, but also our place in society – our role, our rank, and our reputation. This is especially true if ‘society’ is taken in the more restrictive sense of the social groups we belong to, i.e. our family, friends, colleagues, peers, and so on. The specific interaction we have with each of those groups reveals a specific facet of our social self, which has as many referents as persons we get involved with as siblings, workers, or fellows.

Although interoceptive signals are private and almost invisible to others, a growing body of evidence is revealing how interoception shapes the social self in a diverse range of circumstances, often to a surprising extent. For example, interoceptive signals can directly prevent the social self from being confused with the identity of someone else—a distinction which is key to a well-balanced social life (Decety & Sommerville, 2003). In particular, a recent study by Babo-Rebelo et al. (2019) revealed that cardiac signals contribute to encoding the difference between self and other, as imagining oneself in a first-person perspective induced different heartbeat-evoked responses (HERs) at the level of precuneus and posterior cingulate cortex with respect to imagining a friend in a third-person perspective.

Besides shaping the social self as a whole, interoceptive signals can also increase or reduce the chance that specific social properties are included in one’s self-concept, i.e. the chance that one thinks of oneself as socially anxious, afraid of being judged by others, accommodating to injustice, and so forth. People who are more attuned to interoceptive signals are also more likely to consider themselves as socially anxious (Oldroyd et al., 2019; Pollatos et al., 2007; Stevens et al., 2011; see also Terasawa et al., 2013; but cf. Krahé et al., 2018). On the contrary, individuals who have a physiologically higher resting blood pressure, which is linked to a reduced interoception of physical pain (see Makovac et al., 2020), also report to feel less social pain, since they are less likely to describe themselves as fearful of receiving negative evaluations or worried about social rejection (Inagaki et al., 2018; on the role of pain as a threat to the social self, see also Karos et al., 2018). Poor interoception is also linked to high levels of self-reported avoidant attachment (Oldroyd et al., 2019) as well as to several impairments affecting the social self, such as those characterising autism (Quattrocki & Friston, 2014; but see Shah et al., 2016), eating disorders (Ambrosecchia et al., 2017), and conscious vicarious pain/mirror-pain synaesthesia (Bowling et al., 2019).

In turn, social interactions can influence the processing of internal physiological signals. In particular, unbalanced economic offers reduce the perception of pain (Mancini et al., 2014; Nicolardi et al., 2020). Furthermore, direct gaze increases interoceptive accuracy (Isomura & Watanabe, 2020), while social exclusion decreases it (Durlik & Tsakiris, 2015). Thus, the link between interoception and the social self is bi-directional, suggesting a feedback cycle in which inner physiology significatively influences who we think we are when we interact with others, but is also influenced by top-down social cues. As we transition to a world in which traditional social interactions involving the physical presence of the agents are increasingly supplemented or replaced with digital or virtual interactions, a key challenge will be to preserve as much bodily information as possible in the new virtual contexts in order not to disrupt the feedback cycle between interoception and the social self (Monti & Aglioti, 2018).

## Interoceptive constraints on the spiritual self

The concept of self does not include only one’s body and social role; our notion of who we are usually extends also to what James (1890) calls our *spiritual self*, that is, our “inner or subjective being”, our “psychic faculties or dispositions”. Among these faculties, James lists cognitive skills, morality, and agency, remarking that “[t]hese psychic dispositions are the most enduring and intimate part of the self, that which we most verily seem to be (James, 1890, p. 296)”. Indeed, ﻿moral traits are so essential to the self that, if they change or disappear from an individual, people are more likely to deem that she is no longer herself than when she changes her appearance, perceptions, or personality (Strohminger & Nichols, 2014; Strohminger et al., 2017).

Internal physiological signals shape, or misshape, also this part of the self-concept. In particular, personality types that belong to the Dark triad (e.g., Narcissism, Machiavellianism, and Psychopathy) feature not only a socially malevolent character and tendencies toward self-promotion (Paulhus & Williams, 2002), but also anomalous patterns of body awareness. Specifically, Machiavellians have little trust in their own bodily sensations, psychopaths report to be only feebly aware of their interoceptive feelings, and narcissists report an increased interoceptive awareness, more trust in bodily signals, and higher attention regulation abilities (Lyons & Hughes, 2015). More generally, interoception can impact on how one perceives one’s own moral temper and character. Indeed, people who are better at detecting their cardiac beats are also more likely to believe they are better at regulating their affective states, as they self-report to be more inclined to reappraise negative situations (Füstös et al., 2013; Pollatos et al., 2015), suppress their emotions (Pollatos et al., 2015), swiftly and flexibly adapt their emotions to environmental changes (Shaw et al., 2018), differentiate among affective states, and temper their impatience (Weiss et al., 2014; cf. Jäger et al., 2012). At the same time, low interoception has been associated not only with low self-regulation, but also with low resilience (Haase et al., 2016).

Surprisingly, the role of interoceptive signals in what contemporary readers would more naturally call ‘spiritual’ self, i.e. one’s religious identity and attitude towards transcendence, has never been subject to thorough empirical investigations. A partial exception is the neuroscientific study of meditation. Straddling the border between the religious and secular world, meditation is a set of diverse techniques that often rely on conscious monitoring of respiration and bodily feelings in general (Gibson, 2019). The prolonged exercise of such techniques promotes changes in the concept of self, reducing self-judgment, increasing self-centredness, self-directedness, cooperativeness and compassion, and inducing a heightened religious/spiritual self-representation (Campanella et al., 2014; Crescentini, Urgesi, et al., 2014b; Fiori et al., 2017; Neff, 2016; Neff & Germer, 2013; for a review, see Crescentini & Capurso, 2015). Although a consistent finding across the literature is that meditation alters the structural and functional properties of the insular cortex, a key hub of the interoceptive and self-related brain networks (Craig, 2009), the existence, strength, and direction of a causal link between meditation and interoception is far from clear (Gibson, 2019; Khalsa et al., 2020). However, many common explicitly religious practices, from fasting to iterative prayers, tap into the conscious regulation of interoceptive signals such as hunger and respiration to achieve a state of higher spiritual awareness. Based on previous data on the environmental, sensory and neural underpinnings of religion and spirituality (see Crescentini, Aglioti, et al., 2014a; Crescentini et al., 2014b, 2015; Urgesi et al., 2010), a recent theoretical model of the neurobiology of mysticism predicts that during self-transcendent experiences interoceptive inputs should be substantially attenuated or decoupled from exteroceptive signals (van Elk & Aleman, 2017). To test these and other related predictions, new empirical studies may systematically explore or manipulate the relation between active downregulation of physiological signals, interoceptive accuracy, and self-transcendence.

## Interoception and the pure ego

So far, we have shown that changes in the intensity, frequency, and conscious perception of inner physiological signals covary with changes in the likelihood that certain properties are included in one’s notion of oneself or not. In so doing, we have focused on the concept of self as a bundle of self-properties—properties that someone consciously ascribes to their own person. However, the act of ascribing these properties implies that there is an agent who ascribes them to the self. In principle, the self as agent is not identical with the self as bundle of properties. Building on this, James (1890) asserts that the self-agent (which he calls “pure Ego”, or “I”) is the thinking subject that recognises the self-bundle (which he calls “Me”) as an object that keeps existing and being the same across time—regardless of the fact that this continuity is true or illusory. Does interoception shape only the notion of the “Me” or also the notion of the “I”?

Sparse but tantalising evidence points to the fact that interoception can indeed inform also one’s concept of oneself as a thinking and acting subject. Although the precise interplay between interoception and meditation is not firmly established (see above), meditators report that their training allows them to experience the self not as a static being that keeps existing in the same way across time but in an event-like manner, as if a temporary “Me” was created and dissolved with the ebb and flow of desires and impulses (Olendzki, 2006; cf. Farb et al., 2007 and Gibson, 2019). By definition, this conscious ‘loss of selfhood’ cannot take place in the “Me”; rather, it is the “I” that experiences it as a consequence of an interoception-based meditation. Moreover, the experience of ‘losing oneself’, so that the common-sense distinction between the self and the world does not make sense anymore, occurs also in several psychiatric disorders, notably depersonalisation–derealisation (DD), which is characterized by impairments in self-awareness, feelings of disembodiment, and emotional numbing. A single case-study (Sedeño et al., 2014) showed that DD is indeed characterized by altered interoception both at the behavioural and neural level. In a similar vein, another experiment found that, relative to baseline, heartbeat-evoked potentials (HEPs) recorded during a heartbeat counting task become more prominent in controls but not in DD patients (Schulz et al., 2015). However, another study found that DD patients’ cardioceptive accuracy does not significantly differ from controls’ (Michal et al., 2014). This suggests that these patients might have difficulties in integrating visceral signals in their own representation, rather than difficulties in interoceptive awareness per se. At the same time, there is preliminary evidence indicating that interoceptive exposure exercises like hyperventilation may trigger depersonalisation/derealisation experiences in a non-clinically anxious sample (Lickel et al., 2008). More research is needed to sift through these conflicting pieces of evidence and also to assess whether there are impaired interoceptive patterns in other neuro-psychiatric conditions, such as the Cotard syndrome, which is characterised by nihilistic delusions of non-existence or immortality (Berrios & Luque, 1995).

More straightforwardly, a series of clever experiments (Babo-Rebelo, Richter, et al., 2016a; Babo-Rebelo, Wolpert, et al., 2016b) sought to tease apart the visceral influences on the “I” and the “Me” dimensions of the self combining magnetoencephalography (MEG), heartbeat signals, and mind-wandering. In the first experiment of the series, participants mind-wandered while their MEG responses to heartbeats were monitored. At random intervals, they were interrupted by a visual signal and asked to rate how much the interrupted thought included the self as an agent (“I”) or as an object (“Me”). Results showed that i) the more the thought was related to the “I”, the stronger the neural responses to heartbeats in the ventral precuneus; ii) the more the thought had to do with the “Me”, the stronger the neural responses to heartbeats in the ventromedial prefrontal cortex Babo-Rebelo et al., 2016a). A subsequent study confirmed and refined these results through intracranial electroencephalography (iEEG) and suggested that also the right anterior insula may encode the “I” dimension of the self (Babo-Rebelo et al., 2016b).

## Interoception as a firm foundation of self

**Fig. 1:**
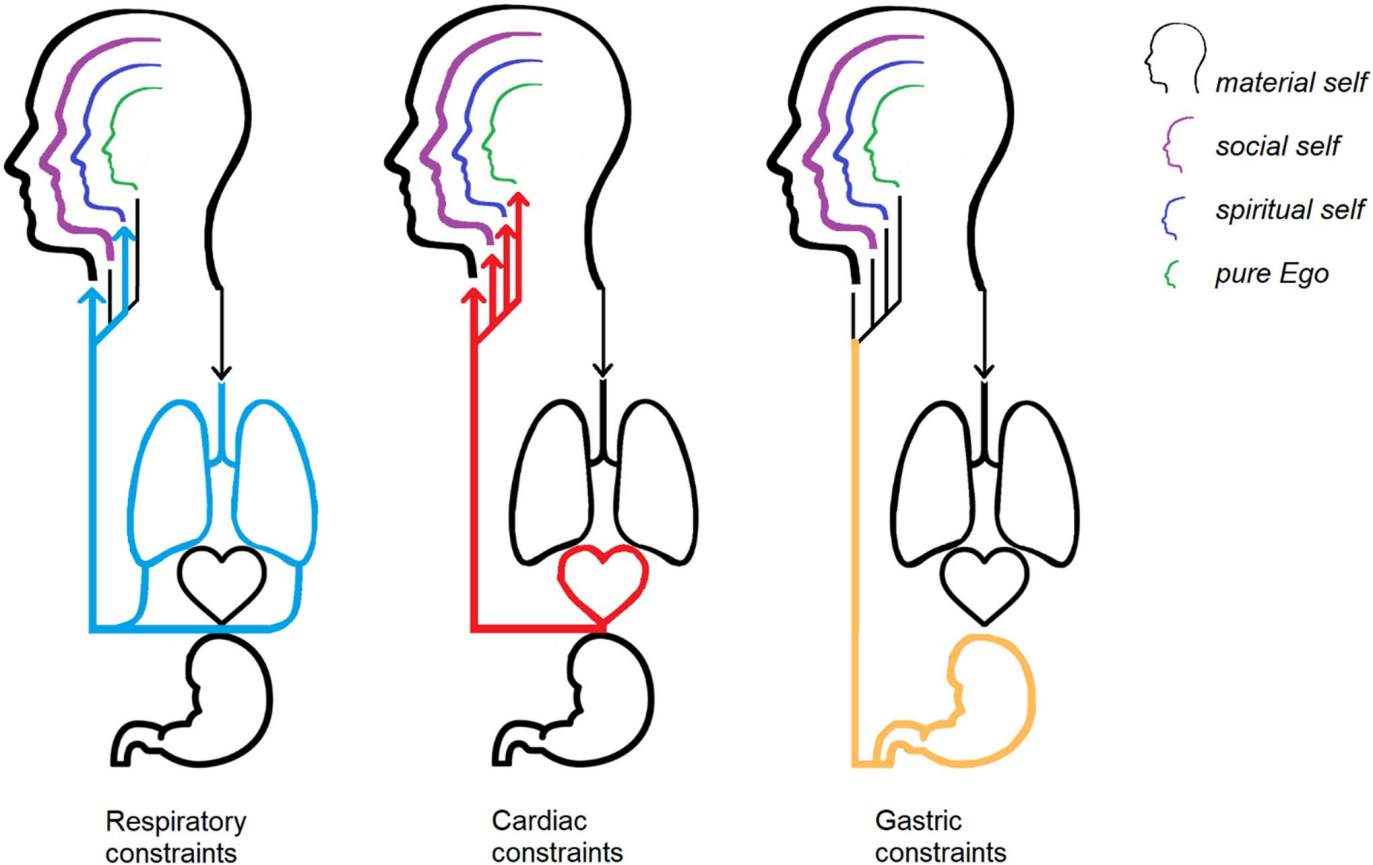
Interoceptive constraints on each dimension of the concept of self, conceived as “Me” (material, social and spiritual self) and as “I” (pure Ego). Coloured arrows show the known influences of specific interoceptive signals on specific facets of the self-concept. Truncated lines with no terminal arrow indicate hypothetical links between the physiological and conceptual realms that are yet to be investigated

Overall, the studies reviewed in the previous sections indicate that inner physiological signals exert a remarkable influence on the material, social, and spiritual facets of the abstract concept of self in its “Me” instance. There is also preliminary evidence linking interoception and the “I” (Fig. 1). Across all levels of analysis, a common thread is the fact that the most intimate, unique, unchanging features of our selves seem to be those which are, quite literally, closest to our heart, i.e. most influenced and shaped by interoceptive signals. On the contrary, extrinsic, negotiable, transient features have a looser link with interoceptive signals. Thus, we claim that interoception provides the self-concept with a firm foundation, contributing to its stability and sanity over time by making it less permeable to external influences.

Insofar as the *material* self is concerned, a number of authors have already underscored that those who are better at detecting cardiac and respiratory signals are also those who are less prone to bodily illusions – the fact that they are more attuned to their bodily signals translates in a greater stability of their material “Me” (Monti et al., 2020; Tsakiris et al., 2011). Interestingly, a recent study revealed that individuals who have a fuzzier concept of self are also more susceptible to the rubber hand and body swap illusion (Krol et al., 2019). Hence, it would be useful to assess whether the relationship between self-concept clarity and bodily illusion proneness is mediated by individual levels of interoceptive accuracy, sensibility, and awareness. If so, this would further corroborate our hypothesis that interoception stabilises the concept of self.

We argue that this stabilising role of interoception on the self-concept is not limited to the material self, but extends also to the *social* and *spiritual* self. As we have seen above, those who are better at detecting interoceptive signals describe themselves as more self-disciplined, more resilient, and more self-centred, as they cope better with social exclusion, tend to prioritise their own needs over those of the others, and are more prone to act selfishly. While interoception-driven self-centredness may easily degenerate into egoism, we note that social interactions can in turn modulate interoceptive abilities (for example, through eye contact) and thus make people more or less centred on their own bodily signals.

Further studies should also better clarify if interoception stabilises not only the “Me” but also the “I” part of the self-concept—for example, measuring how much interoceptive accuracy predicts the degree to which one thinks of oneself as a subject endowed with freedom and continuity over time. Importantly, these signals should not be looked for only in the cardiac domain. For example, a recent study found that spontaneous initiation of action in the classic Kornhuber and Libet tasks is modulated by the phase of the respiratory cycle (Park et al., 2020). Although in that study participants did not report to be aware of any link between respiration and their choice to act, it would be interesting to see if individuals more attuned to respiratory signals also feel to be more in control of their actions and are more inclined to portray themselves as free agents.

If we are right in claiming that interoception serves as a firm foundation for the concept of self, preventing it from becoming too overstretched or too unstable, then two consequences follow. The first is that the classic distinction between ‘core’ and ‘peripheral’ self-conceptions (Gergen, 1968; see Markus & Wurf, 1987) is more than a metaphor—core features of the self-concept are indeed rooted in core physiological signals, whereas marginal or ephemeral features attached to the self are not viscerally coloured. Since James (1890), self-concept theorists have been adding ever new layers and partitions to the concept of self, while struggling to find principles of unity and organisation (Stryker, 1989). Interoception may be one of these principles. The second consequence is that there is now further support to the idea that the self-concept is embodied, rather than purely propositional (Schubert & Koole, 2009). Indeed, the evidence reviewed so far points to an even stronger conclusion, namely, that the self-concept is not just embodied, but deeply embodied, as it is based not only on sensorimotor states (Schubert & Koole, 2009), but also on visceral physiological states. While at the present stage we are not able to grasp the details of the process by which sensory, motor and visceral information translates into linguistic descriptions of the self, we can state that whenever we think of ourselves, we think with our body and not just with our mind.

## References

[CR1] Adler D, Herbelin B, Similowski T, Blanke O (2014). Breathing and sense of self: Visuo–respiratory conflicts alter body self-consciousness. Respiratory Physiology & Neurobiology.

[CR2] Allard E, Canzoneri E, Adler D, Morélot-Panzini C, Bello-Ruiz J, Herbelin B, Blanke O, Similowski T (2017). Interferences between breathing, experimental dyspnoea and bodily self-consciousness. Scientific Reports.

[CR3] Ambrosecchia M, Ardizzi M, Russo E, Ditaranto F, Speciale M, Vinai P, Todisco P, Maestro S, Gallese V (2017). Interoception and Autonomic Correlates during social interactions. Implications for anorexia. Frontiers in Human Neuroscience.

[CR4] Ardizzi M, Ambrosecchia M, Buratta L, Ferri F, Peciccia M, Donnari S, Mazzeschi C, Gallese V (2016). Interoception and positive symptoms in schizophrenia. Frontiers in Human Neuroscience.

[CR5] Aspell JE, Heydrich L, Marillier G, Lavanchy T, Herbelin B, Blanke O (2013). Turning body and self inside out: Visualized heartbeats alter bodily self-consciousness and tactile perception. Psychological Science.

[CR6] Babo-Rebelo M, Richter CG, Tallon-Baudry C (2016). Neural responses to heartbeats in the default network encode the self in spontaneous thoughts. Journal of Neuroscience.

[CR7] Babo-Rebelo M, Wolpert N, Adam C, Hasboun D, Tallon-Baudry C (2016). Is the cardiac monitoring function related to the self in both the default network and right anterior insula?. Philosophical Transactions of the Royal Society B: Biological Sciences.

[CR8] Babo-Rebelo M, Buot A, Tallon-Baudry C (2019). Neural responses to heartbeats distinguish self from other during imagination. NeuroImage.

[CR9] Berlucchi G, Aglioti S (1997). The body in the brain: Neural bases of corporeal awareness. Trends in Neurosciences.

[CR10] Berlucchi G, Aglioti SM (2010). The body in the brain revisited. Experimental Brain Research.

[CR11] Berrios GE, Luque R (1995). Cotard’s syndrome: Analysis of 100 cases. Acta Psychiatrica Scandinavica.

[CR12] Björnsdotter M, Morrison I, Olausson H (2010). Feeling good: On the role of C fiber mediated touch in interoception. Experimental Brain Research.

[CR13] Blanke O, Arzy S (2005). The out-of-body experience: Disturbed self-processing at the temporo-parietal junction The Neuroscientist: A Review. Journal Bringing Neurobiology, Neurology and Psychiatry.

[CR14] Blanke O, Slater M, Serino A (2015). Behavioral, neural, and computational principles of bodily self-consciousness. Neuron.

[CR15] Borghi AM, Barca L, Binkofski F, Tummolini L (2018). Varieties of abstract concepts: Development, use and representation in the brain. Philosophical Transactions of the Royal Society B: Biological Sciences.

[CR16] Botvinick M, Cohen J (1998). Rubber hands “feel” touch that eyes see. Nature.

[CR17] Bowling NC, Botan V, Santiesteban I, Ward J, Banissy MJ (2019). Atypical bodily self-awareness in vicarious pain responders. Philosophical Transactions of the Royal Society B.

[CR18] Campanella F, Crescentini C, Urgesi C, Fabbro F (2014). Mindfulness-oriented meditation improves self-related character scales in healthy individuals. Comprehensive Psychiatry.

[CR19] Carey M, Crucianelli L, Preston C, Fotopoulou A (2021). The role of affective touch in whole-body embodiment remains equivocal. Consciousness and Cognition.

[CR20] Connell L, Lynott D, Banks B (2018). Interoception: The forgotten modality in perceptual grounding of abstract and concrete concepts. Philosophical Transactions of the Royal Society B: Biological Sciences.

[CR21] Craig AD (2002). How do you feel? Interoception: the sense of the physiological condition of the body. Nature Reviews Neuroscience.

[CR22] Craig AD (2009). How do you feel — now? The anterior insula and human awareness. Nature Reviews Neuroscience.

[CR23] Crescentini C, Capurso V (2015). Mindfulness meditation and explicit and implicit indicators of personality and self-concept changes. Frontiers in Psychology.

[CR24] Crescentini C, Aglioti SM, Fabbro F, Urgesi C (2014). Virtual lesions of the inferior parietal cortex induce fast changes of implicit religiousness/spirituality. Cortex.

[CR25] Crescentini C, Urgesi C, Campanella F, Eleopra R, Fabbro F (2014). Effects of an 8-week meditation program on the implicit and explicit attitudes toward religious/spiritual self-representations. Consciousness and Cognition.

[CR26] Crescentini C, Di Bucchianico M, Fabbro F, Urgesi C (2015). Excitatory stimulation of the right inferior parietal cortex lessens implicit religiousness/spirituality. Neuropsychologia.

[CR27] Critchley HD, Harrison NA (2013). Visceral influences on brain and behavior. Neuron.

[CR28] Critchley M (1979). The divine banquet of the brain and other essays.

[CR29] Crucianelli L, Metcalf NK, Fotopoulou AK, Jenkinson PM (2013). Bodily pleasure matters: Velocity of touch modulates body ownership during the rubber hand illusion. Frontiers in Psychology.

[CR30] Decety J, Sommerville JA (2003). Shared representations between self and other: A social cognitive neuroscience view. Trends in Cognitive Sciences.

[CR31] Durlik C, Tsakiris M (2015). Decreased interoceptive accuracy following social exclusion. International Journal of Psychophysiology.

[CR32] Eshkevari E, Rieger E, Longo MR, Haggard P, Treasure J (2012). Increased plasticity of the bodily self in eating disorders. Psychological Medicine.

[CR33] Farb NAS, Segal ZV, Mayberg H, Bean J, McKeon D, Fatima Z, Anderson AK (2007). Attending to the present: Mindfulness meditation reveals distinct neural modes of self-reference. Social Cognitive and Affective Neuroscience.

[CR34] Fiori F, Aglioti SM, David N (2017). Interactions between body and social awareness in yoga. Journal of Alternative and Complementary Medicine.

[CR35] Füstös J, Gramann K, Herbert BM, Pollatos O (2013). On the embodiment of emotion regulation: Interoceptive awareness facilitates reappraisal. Social Cognitive and Affective Neuroscience.

[CR36] Gergen KJ, Gordon C, Gergen KJ (1968). Personal consistency and the presentation of self. The self in social interaction.

[CR37] Gibson J (2019). Mindfulness, interoception, and the body: A contemporary perspective. Frontiers in Psychology.

[CR38] Haase L, Stewart JL, Youssef B, May AC, Isakovic S, Simmons AN, Johnson DC, Potterat EG, Paulus MP (2016). When the brain does not adequately feel the body: Links between low resilience and interoception. Biological Psychology.

[CR39] Herbert BM, Pollatos O (2012). The Body in the Mind: On the Relationship Between Interoception and Embodiment. Topics in Cognitive Science.

[CR40] Heydrich L, Blanke O (2013). Distinct illusory own-body perceptions caused by damage to posterior insula and extrastriate cortex. Brain.

[CR41] Hur J-W, Kwon JS, Lee TY, Park S (2014). The crisis of minimal self-awareness in schizophrenia: A meta-analytic review. Schizophrenia Research.

[CR42] Inagaki TK, Jennings JR, Eisenberger NI, Gianaros PJ (2018). Taking rejection to heart: Associations between blood pressure and sensitivity to social pain. Biological Psychology.

[CR43] Isomura T, Watanabe K (2020). Direct gaze enhances interoceptive accuracy. Cognition.

[CR44] Jäger B, Schmid-Ott G, Ernst G, Dölle-Lange E, Sack M (2012). Entwicklung und Validierung eines Fragebogens zu Ich-Funktionen und zur Selbstregulationsfähigkeit (Hannover-Selbstregulations-Inventar, HSRI). Fortschritte der Neurologie · Psychiatrie.

[CR45] James, W. (1890). *The principles of psychology*. New York : Holt. http://archive.org/details/theprinciplesofp01jameuoft. Accessed 27 May 2021

[CR46] Jenkinson PM, Taylor L, Laws KR (2018). Self-reported interoceptive deficits in eating disorders: A meta-analysis of studies using the eating disorder inventory. Journal of Psychosomatic Research.

[CR47] Karos K, de Williams AC, Meulders A, Vlaeyen JWS (2018). Pain as a threat to the social self: A motivational account. Pain.

[CR48] Keizer A, Smeets MAM, Postma A, van Elburg A, Dijkerman HC (2014). Does the experience of ownership over a rubber hand change body size perception in anorexia nervosa patients?. Neuropsychologia.

[CR49] Khalsa SS, Rudrauf D, Hassanpour MS, Davidson RJ, Tranel D (2020). The practice of meditation is not associated with improved interoceptive awareness of the heartbeat. Psychophysiology.

[CR50] Krahé C, von Mohr M, Gentsch A, Guy L, Vari C, Nolte T, Fotopoulou A (2018). Sensitivity to CT-optimal, affective touch depends on adult attachment style. Scientific Reports.

[CR51] Krol SA, Thériault R, Olson JA, Raz A, Bartz JA (2019). Self-Concept clarity and the bodily self: malleability across modalities. Personality and Social Psychology Bulletin..

[CR52] Kuhn MH, McPartland TS (1954). An empirical investigation of self-attitudes. American Sociological Review.

[CR53] Lenggenhager B, Tadi T, Metzinger T, Blanke O (2007). Video ergo sum: Manipulating bodily self-consciousness. Science.

[CR54] Lickel J, Nelson E, Lickel AH, Deacon B (2008). Interoceptive exposure exercises for evoking depersonalization and derealization: A pilot study. Journal of Cognitive Psychotherapy.

[CR55] Lloyd DM, Gillis V, Lewis E, Farrell MJ, Morrison I (2013). Pleasant touch moderates the subjective but not objective aspects of body perception. Frontiers in Behavioral Neuroscience.

[CR56] Longo MR, Schüür F, Kammers MPM, Tsakiris M, Haggard P (2008). What is embodiment? A psychometric approach. Cognition.

[CR57] Lyons M, Hughes S (2015). Feeling me, feeling you? Links between the Dark Triad and internal body awareness. Personality and Individual Differences.

[CR58] Makovac E, Porciello G, Palomba D, Basile B, Ottaviani C (2020). Blood pressure-related hypoalgesia: A systematic review and meta-analysis. Journal of Hypertension.

[CR59] Mancini A, Betti V, Panasiti MS, Pavone EF, Aglioti SM (2014). Perceiving monetary loss as due to inequity reduces behavioral and cortical responses to pain. European Journal of Neuroscience.

[CR60] Markus H, Wurf E (1987). The dynamic self-concept: a social psychological perspective. Annual Review of Psychology.

[CR61] Michal M, Reuchlein B, Adler J, Reiner I, Beutel ME, Vögele C, Schächinger H, Schulz A (2014). Striking discrepancy of anomalous body experiences with normal interoceptive accuracy in depersonalization-derealization disorder. PLoS ONE.

[CR62] Monti A, Aglioti SM (2018). Flesh and bone digital sociality: On how humans may go virtual. British Journal of Psychology.

[CR63] Monti A, Porciello G, Tieri G, Aglioti SM (2020). The “embreathment” illusion highlights the role of breathing in corporeal awareness. Journal of Neurophysiology.

[CR64] Neff KD (2016). The self-compassion scale is a valid and theoretically coherent measure of self-compassion. Mindfulness.

[CR65] Neff KD, Germer CK (2013). A pilot study and randomized controlled trial of the mindful self-compassion program: A pilot and randomized trial of MSC program. Journal of Clinical Psychology.

[CR66] Nicolardi V, Panasiti MS, D’Ippolito M, Pecimo GL, Aglioti SM (2020). Pain perception during social interactions is modulated by self-related and moral contextual cues. Scientific Reports.

[CR67] Oldroyd K, Pasupathi M, Wainryb C (2019). Social antecedents to the development of interoception: Attachment related processes are associated with interoception. Frontiers in Psychology.

[CR68] Olendzki A, Nauriyal DK, Drummond MS (2006). The transformative impact of non-self. Buddhist thought and applied psychological research: Transcending the boundaries.

[CR69] Paladino M-P, Mazzurega M, Pavani F, Schubert TW (2010). Synchronous multisensory stimulation blurs self-other boundaries. Psychological Science.

[CR70] Park H-D, Bernasconi F, Bello-Ruiz J, Pfeiffer C, Salomon R, Blanke O (2016). Transient modulations of neural responses to heartbeats Covary with bodily self-consciousness. Journal of Neuroscience.

[CR71] Park H-D, Barnoud C, Trang H, Kannape OA, Schaller K, Blanke O (2020). Breathing is coupled with voluntary action and the cortical readiness potential. Nature Communications.

[CR72] Park H-D, Tallon-Baudry C (2014). The neural subjective frame: From bodily signals to perceptual consciousness. Phil. Trans. R. Soc. B.

[CR73] Paulhus DL, Williams KM (2002). The dark triad of personality: Narcissism, machiavellianism, and psychopathy. Journal of Research in Personality.

[CR74] Peled A, Ritsner M, Hirschmann S, Geva AB, Modai I (2000). Touch feel illusion in schizophrenic patients. Biological Psychiatry.

[CR75] Petkova VI, Ehrsson HH (2008). If I were you: Perceptual illusion of body swapping. PLoS ONE.

[CR76] Pollatos O, Traut-Mattausch E, Schroeder H, Schandry R (2007). Interoceptive awareness mediates the relationship between anxiety and the intensity of unpleasant feelings. Journal of Anxiety Disorders.

[CR77] Pollatos O, Kurz A-L, Albrecht J, Schreder T, Kleemann AM, Schöpf V, Kopietz R, Wiesmann M, Schandry R (2008). Reduced perception of bodily signals in anorexia nervosa. Eating Behaviors.

[CR78] Pollatos O, Matthias E, Keller J (2015). When interoception helps to overcome negative feelings caused by social exclusion. Frontiers in Psychology.

[CR79] Porciello G, Daum MM, Menghini C, Brugger P, Lenggenhager B (2016). Not that heart-stopping after all: Visuo-cardiac synchrony does not boost self-face attribution. PLoS ONE.

[CR80] Porciello G, Bufalari I, Minio-Paluello I, Di Pace E, Aglioti SM (2018). The ‘Enfacement’ illusion: A window on the plasticity of the self. Cortex.

[CR81] Porciello G, Monti A, Aglioti SM (2018). The Gastric Network: How the stomach and the brain work together at rest. ELife.

[CR82] Quattrocki E, Friston K (2014). Autism, oxytocin and interoception. Neuroscience and Biobehavioral Reviews.

[CR83] Rebollo I, Devauchelle A-D, Béranger B, Tallon-Baudry C (2018). Stomach-brain synchrony reveals a novel, delayed-connectivity resting-state network in humans. ELife.

[CR84] Ronchi R, Park H-D, Blanke O, Vallar G, Coslett HB (2018). Chapter 15—Bodily self-consciousness and its disorders. Handbook of clinical neurology.

[CR85] Schandry R (1981). Heart beat perception and emotional experience. Psychophysiology.

[CR86] Schubert TW, Koole SL (2009). The embodied self: Making a fist enhances men’s power-related self-conceptions. Journal of Experimental Social Psychology.

[CR87] Schulz A, Köster S, Beutel ME, Schächinger H, Vögele C, Rost S, Rauh M, Michal M (2015). Altered patterns of heartbeat-evoked potentials in depersonalization/derealization disorder: Neurophysiological evidence for impaired cortical representation of bodily signals. Psychosomatic Medicine.

[CR88] Sedeño L, Couto B, Melloni M, Canales-Johnson A, Yoris A, Baez S, Esteves S, Velásquez M, Barttfeld P, Sigman M, Kichic R, Chialvo D, Manes F, Bekinschtein TA, Ibanez A (2014). How do you feel when you can’t feel your body? Interoception, functional connectivity and emotional processing in depersonalization-derealization disorder. PLoS ONE.

[CR89] Sel A, Azevedo RT, Tsakiris M (2017). Heartfelt self: Cardio-visual integration affects self-face recognition and interoceptive cortical processing. Cerebral Cortex.

[CR90] Sforza A, Bufalari I, Haggard P, Aglioti SM (2010). My face in yours: Visuo-tactile facial stimulation influences sense of identity. Social Neuroscience.

[CR91] Shah P, Hall R, Catmur C, Bird G (2016). Alexithymia, not autism, is associated with impaired interoception. Cortex.

[CR92] Shaw DJ, Czekóová K, Pennington CR, Qureshi AW, Špiláková B, Salazar M, Brázdil M, Urbánek T (2018). You ≠ me: Individual differences in the structure of social cognition. Psychological Research Psychologische Forschung.

[CR93] Stevens S, Gerlach AL, Cludius B, Silkens A, Craske MG, Hermann C (2011). Heartbeat perception in social anxiety before and during speech anticipation. Behaviour Research and Therapy.

[CR94] Strohminger N, Nichols S (2014). The essential moral self. Cognition.

[CR95] Strohminger N, Knobe J, Newman G (2017). The true self: A psychological concept distinct from the self. Perspectives on Psychological Science.

[CR96] Stryker S, Berger J, Zelditch M, Anderson B (1989). Further developments in identity theory: singularity versus multiplicity of self. Sociological theories in progress: New formulations.

[CR97] Suzuki K, Garfinkel SN, Critchley HD, Seth AK (2013). Multisensory integration across exteroceptive and interoceptive domains modulates self-experience in the rubber-hand illusion. Neuropsychologia.

[CR98] Tajadura-Jiménez A, Tsakiris M (2014). Balancing the “inner” and the “outer” self: Interoceptive sensitivity modulates self-other boundaries. Journal of Experimental Psychology. General.

[CR99] Terasawa Y, Shibata M, Moriguchi Y, Umeda S (2013). Anterior insular cortex mediates bodily sensibility and social anxiety. Social Cognitive and Affective Neuroscience.

[CR100] Thakkar KN, Nichols HS, McIntosh LG, Park S (2011). Disturbances in body ownership in schizophrenia: Evidence from the rubber hand illusion and case study of a spontaneous out-of-body experience. PLoS ONE.

[CR101] Tsakiris M (2008). Looking for myself: current multisensory input alters self-face recognition. PLoS ONE.

[CR102] Tsakiris M, Jimenez A-T, Costantini M (2011). Just a heartbeat away from one’s body: Interoceptive sensitivity predicts malleability of body-representations. Proceedings of the Royal Society B: Biological Sciences.

[CR103] Urgesi C, Aglioti SM, Skrap M, Fabbro F (2010). The Spiritual Brain: Selective Cortical Lesions Modulate Human Self-Transcendence. Neuron.

[CR104] van Elk M, Aleman A (2017). Brain mechanisms in religion and spirituality: An integrative predictive processing framework. Neuroscience & Biobehavioral Reviews.

[CR105] van Stralen HE, van Zandvoort MJE, Hoppenbrouwers SS, Vissers LMG, Kappelle LJ, Dijkerman HC (2014). Affective touch modulates the rubber hand illusion. Cognition.

[CR106] Villani C, Lugli L, Liuzza MT, Nicoletti R, Borghi AM (2021). Sensorimotor and interoceptive dimensions in concrete and abstract concepts. Journal of Memory and Language.

[CR107] Weiss S, Sack M, Henningsen P, Pollatos O (2014). On the interaction of self-regulation, interoception and pain perception. Psychopathology.

